# Kombinierte intrapulmonale/intramediastinale K-Draht-Lage nach K-Draht-Osteosynthese an der Clavicula: Ein Case Report

**DOI:** 10.1007/s00113-022-01217-5

**Published:** 2022-07-14

**Authors:** Yasmin Youssef, Peter Melcher, Matthias Steinert, Isabella Metelmann, Pierre Hepp, Jan Theopold

**Affiliations:** 1 Klinik für Orthopädie, Unfallchirurgie und Plastische Chirurgie, Universitätsklinikum, Bereich für arthroskopische und spezielle Gelenkchirurgie/Sportverletzungen Leipzig, Liebigstr. 20, 04103 Leipzig, Deutschland; 2 Klinik für Viszeral‑, Thorax‑, Transplantations- und Gefäßchirurgie, Bereich Thoraxchirurgie, Universitätsklinikum Leipzig, Liebigstr. 20, 04103 Leipzig, Deutschland

**Keywords:** Laterale Clavicula-Fraktur, Pseudarthrose, Dislokation, Komplikation, Operative Behandlung, Lateral clavicle fracture, Pseudoarthrosis, Dislocation, Complication, Operative treatment

## Abstract

**Hintergrund:**

Laterale Clavicula-Frakturen können abhängig von der Klassifikation sowohl konservativ oder operativ behandelt werden. Für die operative Versorgung sind verschiedene Operationstechniken beschrieben. Die Wahl einer Operationstechnik ist für das funktionelle Outcome und für einen komplikationsarmen Heilungsverlauf ausschlaggebend.

**Fallbeschreibung:**

Vorgestellt wird der Fall einer Patientin mit einer sekundären Dislokation zweier K‑Drähte nach K‑Draht-Osteosynthese. Im Rahmen einer sekundären Dislokation kam es zu einem Wandern des Drahtes in das Mediastinum und das Lungengewebe direkt unter den Aortenbogen. Zur Verhinderung weiterer Verletzungen konnte das chirurgische Fremdmaterial über eine uniportale videoassistierte Thorakoskopie geborgen werden.

**Zusammenfassung:**

Bei der Versorgung von lateralen Clavicula-Frakturen sollte auf eine instabile K‑Draht-Osteosynthese unbedingt verzichtet werden. Bei Vorliegen von sicheren Operationsverfahren (Plattenosteosynthese, Hybridversorgungen) sollten diese bevorzugt werden. Bei Durchführung primärer oder additiver K‑Draht-Osteosynthesen ist auf deren Sicherung z. B. durch Umbiegen zu achten, da es bei fehlerhafter Versorgung zu erheblichen Komplikationen, wie Pseudarthrose oder sekundären Dislokation des Materials, kommen kann.

## Abstract (en)

**Background:**

Lateral clavicle fractures can be treated both conservatively and surgically depending on the fracture classification. Different surgical techniques have been described for the operative treatment. The selection of the appropriate technique is decisive for the functional outcome and healing process without complications.

**Case:**

We report on a patient with a secondary dislocation of two Kirschner wires after Kirschner wire osteosynthesis. The secondary dislocation caused one of the wires to migrate into the mediastinum and pulmonary tissue, directly under the aortic arch. To prevent further migration with potential damage to surrounding structures, a uniportal video-assisted thoracoscopy was performed to retrieve the wire.

**Conclusion:**

The treatment of lateral clavicle fractures should be performed with bent Kirschner wires as they can otherwise lead to severe complications including the occurrence of pseudarthrosis or secondary migration of the material. Safe and stable surgical techniques (plate osteosynthesis, hybrid treatment) should be preferred if they are available.

## Einleitung

Clavicula-Frakturen machen 2,6–4,0 % aller Frakturen bei Erwachsenen und 44 % der Frakturen im Bereich des Schultergürtels aus [1]. Männer haben im Vergleich zu Frauen ein doppelt so hohes Risiko, sich eine Clavicula-Fraktur zuzuziehen [1, 2]. Clavicula-Frakturen weisen in der Prävalenz zwei Häufigkeitsgipfel, im jungen Erwachsenenalter und bei älteren Patienten (> 70 Jahre), auf [3].

Häufige Ursachen sind direkte Stürze auf die Schulter und Verkehrsunfälle [4]. Die Mehrzahl der Clavicula-Frakturen (69–82 %) erfolgt im mittleren Drittel des Schaftes, gefolgt von denen an der lateralen Clavicula (12–26 %) [4].

Die Anzahl der operativen Behandlungen von Clavicula-Frakturen nimmt weltweit zu, auch bei älteren Patienten [5]. Besonders für dislozierte Frakturen im mittleren Schaftbereich liegen einige vergleichende Studien vor. Die konservative Therapie zeigt sich hier als Goldstandrad mit dem Behandlungsziel eines „guten“ Outcome. Bei höherem Anspruch oder vorliegenden Risikofaktoren wie Nikotinabusus sollte eine operative Therapie jedoch in Betracht gezogen werden [5, 6].

Für die operative Versorgung sind verschiedene Techniken beschrieben worden. Neben der reinen K-Draht-Osteosynthese, wurden auch unterschiedliche Nagel-, Platten- und Hybridosteosynthesen in der Literatur beschrieben. Obwohl mit einer deutlich erhöhten Komplikationsrate versehen, gehört die reine Kirschner(K)-Draht-Osteosynthese immer noch zu den genutzten Verfahren in der operativen Versorgung von lateralen Clavicula-Frakturen [7, 8]. Eine bekannte, besonders schwerwiegende Komplikation stellt die Migration der K‑Drähte dar [9–12].

Ziel dieser Arbeit ist die Verdeutlichung der Risiken instabiler Osteosyntheseverfahren an der Clavicula anhand einer Patientin mit einer außergewöhnlichen Komplikation, verursacht durch eine 2fache K‑Draht-Migration nach K‑Draht-Osteosynthese einer lateralen Clavicula-Fraktur.

## Fallbericht

### Anamnese

Vorgestellt wird eine 85 Jahre alte Patientin, die mit einer sekundären Dislokation eines K‑Drahtes nach operativer Stabilisierung einer lateralen Clavicula-Fraktur (Neer IIIb; Jäger/Breitner IIa) rechts in unser Klinikum zugewiesen wurde.

**Figure 1:**
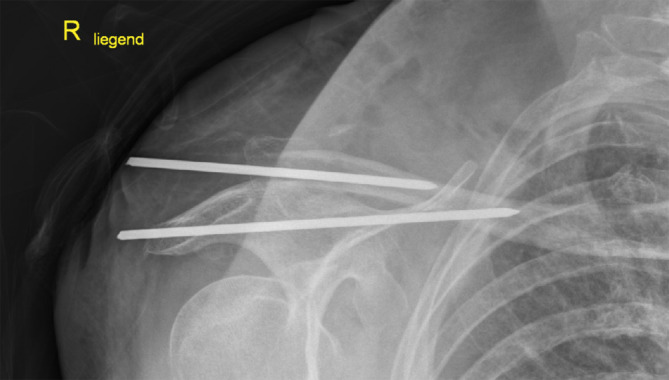

Die primäre operative Versorgung erfolgte in einem externen Krankenhaus mittels direkter K‑Draht-Osteosynthese. Röntgenologisch zeigt sich, dass die Enden der K‑Drähte nicht umgebogen wurden. Zudem kann eine korrekte, intraossäre Lage in beiden Frakturfragmenten nicht sicher belegt werden, da den Autoren nur eine Ebene vorliegt (Abb. 1). Allgemein wies die Patientin folgende Nebendiagnosen auf: arterielle Hypertonie, COPD, Diabetes mellitus und chronische Niereninsuffizienz.

### Befund/Diagnose

Zwei Wochen nach der primären Osteosynthese stellte sich die Patientin mit starken Schmerzen und einer schlecht heilenden Operationsnarbe erneut in der versorgenden Klinik vor. Die Bewegung im betroffenen Schultergelenk war stark Schmerzhaft (VAS 8) und deutlich eingeschränkt. Der Bereich der Operationsnarbe war stark druckdolent, gerötet, überwärmt, und es zeigte sich am lateralen Rand eine Blase. Weitere klinische Untersuchungen zeigten, dass einer der K‑Drähte nach lateral migriert war und sich ein Abszess ausgebildet hatte. In einem operativen Eingriff wurde der Abszess ausgeräumt und der migrierte K‑Draht entfernt. Der zweite K‑Draht wurde in situ belassen.

**Figure 2:**
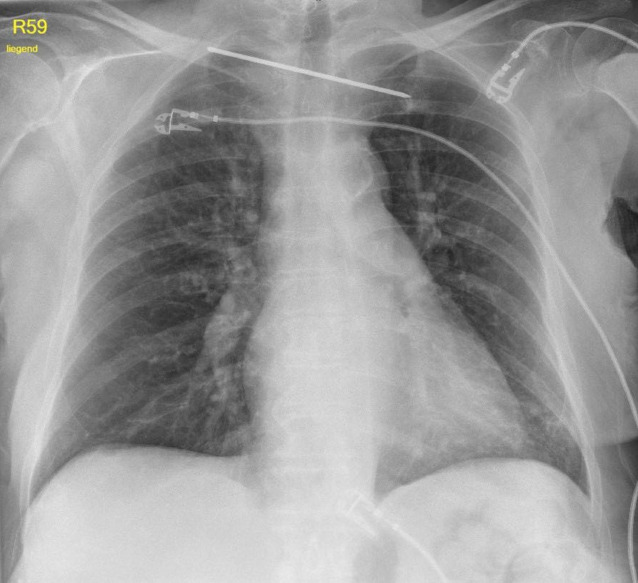

**Figure 3:**
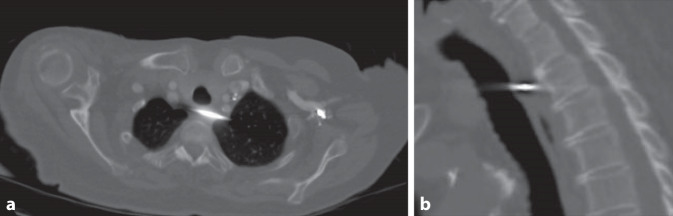

Bei einer Röntgenkontrolle zur Planung der Materialentfernung bei sonst asymptomatischer Patientin zeigte sich eine Verlagerung des verbliebenen K‑Drahtes nach intrathorakal/mediastinal (Abb. 2). Eine auswärts durchgeführte native Computertomographie (CT) bestätigte die Dislokation des K‑Drahtes und zeigte eine Perforation beider Pleurahöhlen und die Migration in das Mediastinum. Der K‑Draht verlief dorsal der A. vertebralis, zwischen Trachea und Ösophagus ohne eine Läsion dieser Strukturen (Abb. 3).

### Therapie und Verlauf

Nachdem die Patientin in unser Klinikum aufgenommen wurde, erfolgte die gemeinsame Therapieplanung mit den Kollegen des Bereichs für Thoraxchirurgie. Diese stellten die Indikation zur geplanten uniportalen videoassistierten Thorakoskopie links. Bei dieser konnte der K‑Draht erfolgreich entfernt werden. Postoperativ bildete sich in diskretes Emphysem im Bereich des linken Hemithorax. Ein Pneumothorax konnte radiologisch ausgeschlossen werden.

**Figure 4:**
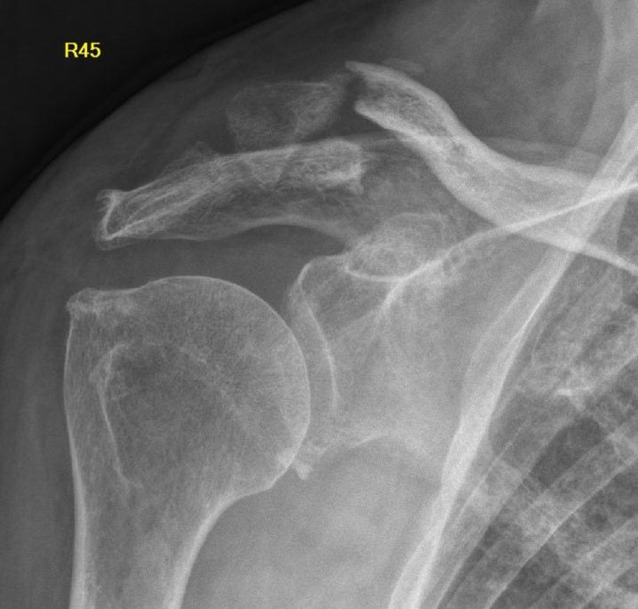

Die radiologische und klinische Verlaufskontrolle der Schulter zeigte weiterhin im Bereich der Fraktur instabile Verhältnisse. Die Patienten berichtete aber nur über leichte Einschränkungen im Alltag bei subjektivem Wohlbefinden. Über der lateralen Clavicula bestand weiterhin ein Druckschmerz. Die Muskelkraft war nicht eingeschränkt und die Sensibilität intakt. Die Bewegungsumfang im Schultergelenk betrug 120° Anteversion, 120° Abduktion und 60° Innen- und Außenrotation. Die radiologische Untersuchung zeigte die bekannte Fraktur mit verzögerter Frakturheilung (Abb. 4). Aufgrund der geringen Beschwerden, des hohen Lebensaltersalters und der bekannten Nebendiagnosen wurde von einer erneuten operativen Versorgung abgesehen. Die Patientin und deren Angehörigen stimmten dem zu. Sechs Monate nach der K‑Draht-Entfernung gibt die Patientin keine relevanten Schmerzen an und fühlt sich subjektiv wohl. Greifen und Faustschluss sind möglich. Eine leichte Einschränkung im Alltag stellt die Mobilität im Schultergelenk dar – Abduktion und Elevation sind nur bis auf Brusthöhe möglich. Mediastinale oder pulmonale Probleme bestanden nicht.

## Diskussion

Frakturen im lateralen Drittel sind seltener als Frakturen der medialen Clavicula [13]. Sie neigen, im Vergleich, jedoch zu mehr Komplikationen, wie z. B. Pseudarthrosen [13]. Am häufigsten befinden sich Pseudarthosen, mit 30-45% im  lateralen Drittel der Clavicula [8]. Die laterale Clavicula-Fraktur kann, je nach Dislokationsgrad, sowohl konservativ als auch operativ versorgt werden. Gute klinische Outcomes konnten in der konservativen Versorgung von nichtdislozierten Clavicula-Frakturen erzielt werden, während Frakturen im Bereich der CC-Bänder eine Pseudarthrosenrate zwischen 30 und 44 % aufweisen [3]. Die Indikation zur operativen Versorgung besteht bei stark dislozierten und offenen Frakturen oder bei vaskulären und neuralen Begleitverletzungen [7, 8]. Für die operative Versorgung sind unterschiedliche Techniken beschrieben [7, 8]. Unter anderem werden in der aktuellen Literatur intramedulläre Schienung, Versorgungen mittels K‑Drähten, TightRope-System, Nagelosteosynthese, Hakenplatte, Plattenosteosynthese, Hybridosteosynthesen und arthroskopisch-navigierte Techniken beschrieben [7, 8]. Ein Goldstandard konnte sich bisher nicht etablieren, sodass die Auswahl der operativen Versorgung in Zusammenschau des klinischen Befundes, des Frakturtyps, des Patientenprofils und des Standards an der versorgenden Einrichtung zu wählen ist.

Einzelne Fallberichte über schwerwiegende Komplikationen, insbesondere durch Drahtmigration nach Versorgung einer lateralen Clavicula-Fraktur mittels K‑Draht existieren bereits [9–12]. Trotz dieser katastrophalen Komplikationen wird diese Technik weiterhin verwendet [7–10]. Neben der im Fall, erneut beschriebenen, Drahtmigration in „innere Organe“, ergibt sich ebenfalls ein schlechteres klinisches Ergebnis durch eine höhere Rate an Pseudarthrosen und Osteolysen im lateralen Frakturfragment [8]. Zur Verdeutlichung der komplexen Komplikationen berichten wir über eine Patientin, bei der die Drähte nach Osteosynthese sekundär zweizeitig disloziert sind. Diese kombinierte mediastinale und intrapulmonale Drahtfehllage stellte für die Patientin eine besondere Gefahrenkonstellation dar. In dem vorgestellten Fall wurde die Migration des Drahtes erleichtert, da die Enden nicht umgebogen wurden. Auch liegt den Autoren keine Bilddokumentation vor, welche die korrekte, intraossäre Lage in beiden Frakturfragmenten beweist, sodass eine bereits primäre Drahtfehllage nicht ausgeschlossen werden kann (Abb. 1). Eine adäquate Versorgung hätte eine röntgenologisch gesicherte K‑Draht-Transfixation des AC-Gelenkes und der Fraktur beinhaltet.

## Fazit für die Praxis

Zusammenfassend sollte, sofern möglich, aufgrund des Vorliegens anderer, sicherer und verlässlicherer Operationsverfahren die Osteosynthese mittels K‑Draht kritisch abgewogen werden. Ist eine andere Operationsmethode nicht anwendbar, sollte der Patient ausführlich über die hohen Risiken einer K‑Draht-Migration aufgeklärt werden. Der K‑Draht sollte am lateralen Enden umgebogen und die korrekte Lage röntgenologisch in 2 Ebenen bewiesen und dokumentiert werden. Es sollten engmaschige Röntgenkontrollen erfolgen und eine frühestmögliche Materialentfernung in Betracht gezogen werden, um eine Dislokation des Materials und damit schwerwiegende Komplikationen zu verhindern.
